# Analysis and validation of serum biomarkers in brucellosis patients through proteomics and bioinformatics

**DOI:** 10.3389/fcimb.2024.1446339

**Published:** 2025-01-13

**Authors:** Xiao Li, Bo Wang, Xiaocong Li, Juan He, Yue Shi, Rui Wang, Dongwei Li, Ding Haitao

**Affiliations:** ^1^ Department of Inner Mongolia Clinical Medicine College, Inner Mongolia Medical University, Hohhot, Inner Mongolia, China; ^2^ Department of Clinical Laboratory Medicine Center, Inner Mongolia Autonomous Region People’s Hospital, Hohhot, Inner Mongolia, China; ^3^ Inner Mongolia Academy of Medical Sciences, Hohhot, Inner Mongolia, China

**Keywords:** brucellosis, biomarkers, proteomics, bioinformatics, differential expression analysis, weighted gene co-expression network analysis (WGCNA), machine learning

## Abstract

**Introduction:**

This study aims to utilize proteomics, bioinformatics, and machine learning algorithms to identify diagnostic biomarkers in the serum of patients with acute and chronic brucellosis

**Methods:**

Proteomic analysis was conducted on serum samples from patients with acute and chronic brucellosis, as well as from healthy controls. Differential expression analysis was performed to identify proteins with altered expression, while Weighted Gene Co-expression Network Analysis (WGCNA) was applied to detect co-expression modules associated with clinical features of brucellosis. Machine learning algorithms were subsequently used to identify the optimal combination of diagnostic biomarkers. Finally, ELISA was employed to validate the identified proteins.

**Results:**

A total of 1,494 differentially expressed proteins were identified, revealing two co-expression modules significantly associated with the clinical characteristics of brucellosis. The Gaussian Mixture Model (GMM) algorithm identified six proteins that were concurrently present in both the differentially expressed and co-expression modules, demonstrating promising diagnostic potential. After ELISA validation, five proteins were ultimately selected.

**Discussion:**

These five proteins are implicated in the innate immune processes of brucellosis, potentially associated with its pathogenic mechanisms and chronicity. Furthermore, we highlighted their potential as diagnostic biomarkers for brucellosis. This study further enhances our understanding of brucellosis at the protein level, paving the way for future research endeavors.

## 1 Introduction

Brucellosis is a zoonotic disease caused by bacteria of the genus *Brucella* invading the body, posing a significant threat to public safety and human health as a shared illness between humans and animals (Franco et al., 2007). Humans can become infected through contact with the secretions of infected animals or carriers, including milk, feces, and urine (Jiang et al., 2020). In the acute phase of the disease, patients often present with symptoms such as fever, sweating, fatigue, and abnormalities in the blood system. In the chronic phase, joint pain and arthritis become predominant symptoms (Liu et al., 2021). Due to the disease’s diverse clinical manifestations and significant individual variations among patients, brucellosis is prone to misdiagnosis and underdiagnosis. This often leads to missed opportunities for optimal treatment and can result in the progression of the disease to a chronic state (Wu et al., 2022). Currently, combination antibiotic therapy is the primary treatment for brucellosis. Diagnosis is typically made by clinicians through a combination of patient history, clinical presentations, and laboratory tests (Shakir, 2021). According to a report by the World Health Organization (WHO), approximately 500,000 new cases of brucellosis are reported annually. However, due to limitations in diagnostic techniques, the actual incidence rate is likely to be much higher (Marvi et al., 2018). Over the past two years, the incidence of brucellosis in Inner Mongolia and surrounding regions has increased exponentially, exhibiting distinct spatiotemporal distribution patterns (Yang et al., 2023a).

Laboratory diagnosis of brucellosis typically relies on bacterial culture and serological immunological tests. Common serological tests include the Rose Bengal Plate Test (RBPT) and the standard tube agglutination test (SAT) (Yagupsky et al., 2019). However, these methods have certain limitations. For example, bacterial culture is time-consuming, which can delay timely disease treatment. Additionally, serological tests are often limited by issues related to sensitivity and specificity, which may lead to misdiagnosis, particularly in the early stages of infection (Di Bonaventura et al., 2021). Serum proteins, which are derived from various tissues and organs, dynamically reflect the body’s physiological processes. Their expression levels can change during the onset and progression of diseases. In many cases, serum protein levels can serve as biomarkers to assist physicians in diagnosing diseases, monitoring their progression, evaluating treatment efficacy, and predicting patient prognosis. With the advancements in mass spectrometry technology, it is now possible to detect and identify low-abundance proteins in serum with high sensitivity and specificity. This has opened up new possibilities for screening and discovering protein biomarkers associated with diseases (Geyer et al., 2017). In this study, we applied Astral-DIA proteomics technology and bioinformatics analysis to serum samples collected from both acute and chronic brucellosis patients, as well as healthy controls. The primary objective was to identify potential protein biomarkers that could effectively differentiate between acute and chronic brucellosis and distinguish brucellosis patients from healthy individuals.

**Figure 1:**
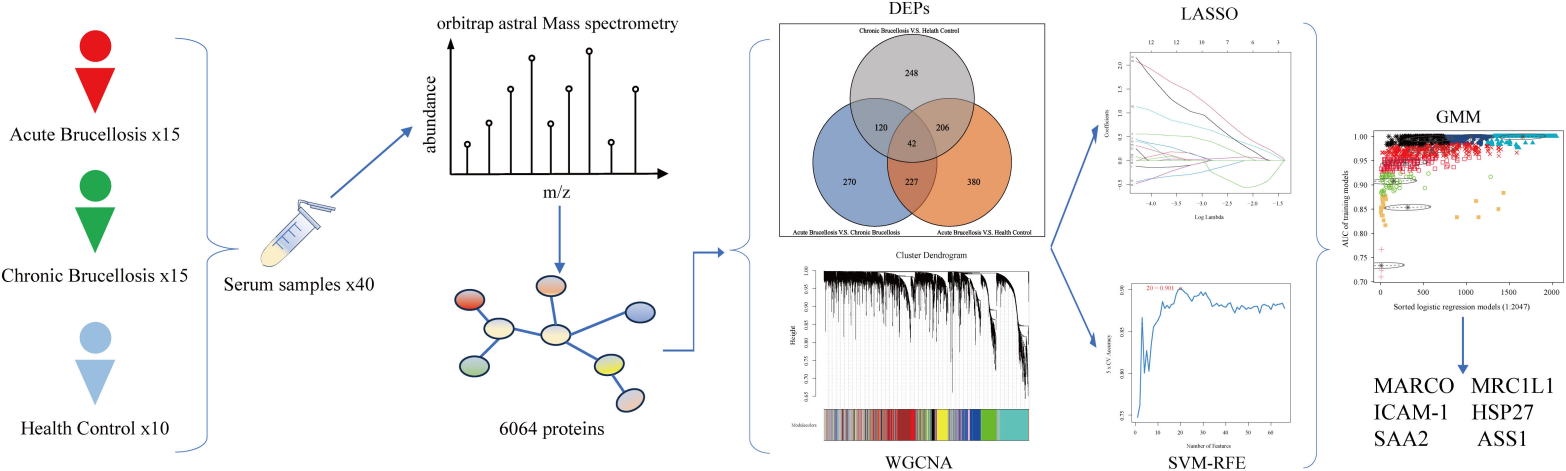
Flowchart of this study.

In this study, we enrolled 40 participants, consisting of 15 acute Brucellosis patients, 15 chronic Brucellosis patients, and 10 healthy controls. Serum samples were collected from all participants, and their protein expression profiles were analyzed using an Orbitrap Astral mass spectrometer. A total of 6,064 proteins were identified, and both differential expression analysis and Weighted Gene Co-expression Network Analysis (WGCNA) were performed on these proteins. The differential expression analysis identified three groups of key differentially expressed proteins: 42 common differentially expressed proteins, 120 proteins related to chronicity, and 227 proteins associated with the acute phase. WGCNA revealed two modules that were significantly correlated with Brucellosis characteristics. Combining the results from both approaches, 69 proteins were identified as common to both analyses. Subsequently, machine learning algorithms were applied to further refine the results, leading to the identification of six proteins that hold potential for improving the diagnosis and understanding of Brucellosis mechanisms. A flowchart summarizing the study design is shown in **Figure 1**.

## 2 Materials and methods

### 2.1 Research populations

A total of 40 participants were included in this study, divided into three groups: 15 individuals with acute Brucellosis, 15 with chronic Brucellosis, and 10 healthy controls. The diagnostic criteria for Brucellosis were as follows: (1) Epidemiological history. (2) Laboratory tests: Isolation of *Brucella* from blood cultures or positive results in both the Rose Bengal Plate Test (RBPT) and the serum agglutination test (SAT) with titers of 1:100++ or higher, or patients with symptoms persisting for more than one year with titers of 1:50++ or higher. (3) Clinical manifestations: Symptoms such as fever, sweating, fatigue, joint pain, and other signs of infection. A diagnosis of Brucellosis is confirmed when all three criteria are met (Liu et al., 2022). (4) Clinical staging: A diagnosis is classified as acute if symptoms occur within 3 months of onset, while cases presenting after more than 6 months are considered chronic (Jiang et al., 2019). (5) Inclusion criteria: Participants meeting the diagnostic criteria with complete and sufficient clinical and laboratory data. (6) Exclusion criteria: Excluding individuals with concurrent infections, immunological disorders, underlying diseases, or abnormalities in the immune system.

### 2.2 Preparation for the removal of high-abundance protein samples

All samples were initially frozen and then transferred to centrifuge tubes. Following the instructions provided in the High-Select™ Top14 Abundant Protein Depletion Resin Kit protocol, high-abundance proteins were removed, and the protein solution was collected. The samples were concentrated to the appropriate volume using a 3 kDa ultrafiltration tube. Subsequently, the samples were exchanged with 8M urea solution (containing a protease inhibitor) three times, with each exchange lasting 40 seconds. The samples were then lysed on ice for 30 minutes, with vortexing every 5 minutes for 5-10 seconds. After lysis, the samples were centrifuged at 12,000g for 30 minutes at 4°C to collect the supernatant. Protein concentration was determined using the bicinchoninic acid (BCA) assay, according to the manufacturer’s instructions. Finally, to assess the efficiency of the high-abundance protein removal, SDS-PAGE electrophoresis was performed.

### 2.3 Protein digestion

A 100μg aliquot of the protein sample was resuspended in 100 mM Triethylammonium bicarbonate buffer (TEAB) to achieve the final concentration. The mixture was reduced by adding 10 mM Tris(2-carboxyethyl) phosphine (TCEP), and the reaction was allowed to proceed at 37°C for 60 minutes. Following reduction, 40 mM iodoacetamide (IAM) was added, and the reaction was incubated in the dark at room temperature for 40 minutes. To precipitate the proteins, pre-chilled acetone was added in a 6:1 (acetone: sample) ratio, and the mixture was incubated at -20°C for 4 hours. The protein pellet was resuspended in 100µL of 100 mM TEAB for thorough dissolution. Trypsin was added at a mass ratio of 1:50 (enzyme: protein), and the mixture was incubated overnight at 37°C for digestion.

### 2.4 Peptide desalting and quantification

After trypsin digestion, the peptides were concentrated using a vacuum pump. The resulting peptides were then re-solubilized in 0.1% trifluoroacetic acid (TFA). Desalting was performed using an HLB (Hydrophilic-Lipophilic Balance) column, and the desalted peptides were further concentrated using a vacuum concentrator. Finally, the peptide concentration was determined using the Thermo Fisher Scientific Peptide Quantification Kit (Item #23275).

### 2.5 DIA mass detection

Based on peptide quantification results, the peptides were analyzed by an Vanquish Neo UHPLC system (Thermo, USA) coupled with an Orbitrap Astral mass spectrometer (Thermo, USA) at Majorbio Bio-Pharm Technology Co. Ltd. (Shanghai, China). Briefly, a uPAC High Throughput column (75 μm × 5.5 cm, Thermo Fisher Scientific, USA) was used for chromatographic separation. The mobile phases consisted of solvent A (water with 2% acetonitrile (ACN) and 0.1% formic acid) and solvent B (water with 80% ACN and 0.1% formic acid). The peptides were eluted using a 180-minute gradient at a flow rate of 500 nL/min.

Data-independent acquisition (DIA) data were collected using an Orbitrap Astral mass spectrometer operating in DIA mode. The detection mode was set to positive ionization, with the ionization source voltage set to 1.5 kV. Both MS and MS/MS data were acquired over a m/z range of 100 to 1700.

### 2.6 Protein identification

Spectronaut™ software (Version 18) was used to analyze the DIA raw data (Zhang et al., 2020). A selection criterion of 6 peptides per protein and 3 daughter ions per peptide was applied for quantitative analysis. The parameters were set as follows: Protein FDR ≤ 0.01, Peptide FDR ≤ 0.01, Peptide Confidence ≥ 99%, and XIC width ≤ 75 ppm. Shared and modified peptides were excluded, and the peak areas were calculated and summed to generate the quantitative results. Only proteins with at least one unique peptide were included in the protein identification.

### 2.7 Data preprocessing and differential analysis

The protein expression matrix was preprocessed using R software (version 4.3.1) and the “SeqKnn” package (version 1.0.1). Initially, proteins with missing values in ≥70% of the samples were removed. Missing values in other proteins were imputed using the SeqKnn method, and the data were then log-transformed. Differential expression analysis was performed using the “limma” package (version 3.56.2) (Ritchie et al., 2015), comparing the acute, chronic, and control groups. Proteins with a |Fold Change| ≥ 1.5 and a p-value ≤ 0.05 were considered significantly upregulated or downregulated. Volcano plots and box plots were generated using the “ggplot2” package (version 3.4.4), and a heatmap was created using the “pheatmap” package (version 1.0.12).

### 2.8 Weighted correlation network analysis

To identify highly correlated gene modules, we performed a Weighted Gene Co-expression Network Analysis (WGCNA). This analysis aimed to explore the relationships between modules and their associations with external traits, such as clinical indicators, to identify potential diagnostic biomarkers with clinical relevance. The method is also applicable to proteomics research (Wu et al., 2020). We used the “WGCNA” package (version 1.72.1) to construct the co-expression network (Langfelder and Horvath, 2008). First, we conducted a clustering analysis to identify potential outlier samples. We then used the R function “pickSoftThreshold” to determine the optimal soft-thresholding power parameter (β). The R function “blockwiseModules” was subsequently applied to construct the co-expression network automatically, with parameter settings: minModuleSize = 30 and mergeCutHeight = 0.25. Next, we calculated the correlation between different modules and external traits, followed by the generation of a correlation heatmap. The module with the highest correlation to the traits of interest was selected as the key module, and the proteins within this module were used for further feature protein selection. Gene significance (GS) and module membership (MM) were computed, and a scatter plot was generated. Finally, proteins with the strongest connections in the key module were exported to Cytoscape (version 3.9.1) for network visualization (Shannon et al., 2003).

### 2.9 Enrichment analysis of characteristic genes

Gene Ontology (GO) and Kyoto Encyclopedia of Genes and Genomes (KEGG) pathway analyses were performed on the feature proteins using the Database for Annotation, Visualization, and Integrated Discovery (DAVID) (https://david.ncifcrf.gov) (Huang et al., 2007). A p-value ≤ 0.05 was considered statistically significant.

### 2.10 Machine learning

We employed three machine learning algorithms—LASSO, SVM-RFE, and Gaussian Mixture Model (GMM)—to identify the most valuable biomarkers for diagnosing Brucellosis. LASSO (Least Absolute Shrinkage and Selection Operator) regression is used to select variables by finding the optimal λ that minimizes classification errors. During the fitting process, LASSO automatically conducts variable selection, making it ideal for feature screening when constructing optimal classification models. The analysis was performed using the “glmnet” package (version 4.1.8) (Engebretsen and Bohlin, 2019). SVM-RFE (Support Vector Machine-Recursive Feature Elimination) is a feature selection method that iteratively eliminates features based on their importance. Starting with all features, it evaluates each feature’s score using model training samples and removes the features with the lowest scores based on model performance. The remaining features are then used to train the model for the next iteration. This process continues until the most significant features are identified, and model performance is evaluated using cross-validation (Huang et al., 2017). The SVM-RFE analysis was conducted using the “e1071” package, with K-Fold Cross-Validation (k = 5). Gaussian Mixture Model (GMM) is a probabilistic unsupervised clustering technique that partitions data variables into a mixture of multiple Gaussian distributions. This approach aids in modeling the data more flexibly and selecting optimal variable combinations. The GMM analysis was carried out using the “mclust” package (version 6.0.0) (Scrucca et al., 2016), and the optimal variables were selected based on the magnitude of their Area Under the Curve (AUC) values.

### 2.11 Enzyme-linked immunosorbent assay

The potential diagnostic protein biomarkers were further validated in a larger cohort using the following ELISA kits: Human Serum Amyloid A2 (SAA2 GENLISA™ ELISA Kit, item #KBH6164), Human Argininosuccinate Synthase (ASS1 GENLISA™ ELISA Kit, item #KBH4985), Human MARCO (MARCO ELISA Kit, item #EK2242 and item #EK2049), Human sICAM-1/CD54 (sICAM-1/CD54 ELISA Kit, item #EK189-96), and Human HSP27/HSPB1 (HSP27/HSPB1 ELISA Kit, item #EK1244-96). The cohort included 30 acute-phase patients, 30 chronic-phase patients, and 20 healthy controls, totaling 80 participants, of whom 40 patients were also included in the proteomics analysis.

## 3 Results

### 3.1 Screening and biological function enrichment analysis of differential proteins in acute and chronic brucellosis

**Figure 2:**
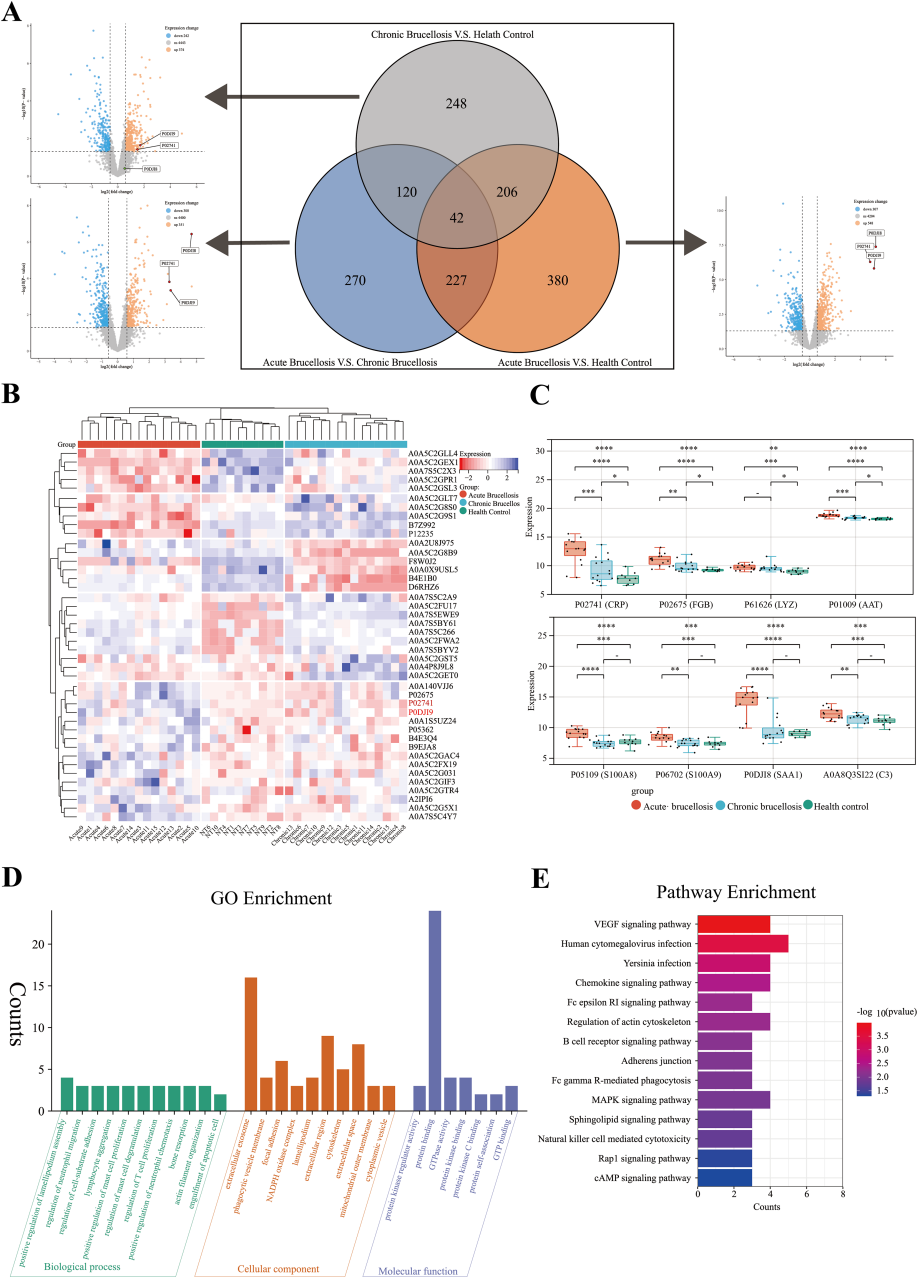
Differential protein expression and chronic protein enrichment analysis in three groups. **(A)** A volcano plot and a Venn diagram collectively depict the differential protein expression among three groups in pairwise comparisons. **(B)** A heatmap illustrates the expression profiles of the 42 differentially expressed proteins in serum samples from the three groups. **(C)** The box plot shows the expression levels of acute-phase proteins in serum samples from the three groups. **(D)** Gene Ontology (GO) enrichment analysis of the 120 proteins potentially associated with the chronicity of Brucellosis. **(E)** Kyoto Encyclopedia of Genes and Genomes (KEGG) enrichment analysis of the 120 proteins potentially associated with the chronicity of Brucellosis.

After mass spectrometry analysis, a total of 6064 proteins were identified. After removing proteins with missing values greater than or equal to 70%, 5060 proteins remained for further analysis. Differential expression analysis was conducted using the “limma” package in R software. The differentially expressed proteins across the three groups were visualized using volcano plots, and the overlap of these proteins among the groups was depicted with Venn diagrams, as shown in **Figure 2A**. A total of 1494 proteins exhibited differential expression across the three groups. Among these, 248 proteins were differentially expressed only between the chronic Brucellosis group and the healthy control group, while 270 proteins were differentially expressed exclusively between the acute Brucellosis group and the chronic Brucellosis group. Additionally, 380 proteins showed differential expression solely between the acute Brucellosis group and the healthy control group. Furthermore, 42 proteins were differentially expressed in all three groups, indicating their potential association with *Brucella* infection. A subset of 120 proteins showed consistent differential expression between the acute Brucellosis group and the chronic Brucellosis group, as well as between the chronic Brucellosis group and the healthy controls. These proteins may play a role in the progression from acute to chronic Brucellosis (Yang et al., 2023b), as illustrated in **Figure 2A**. Lastly, we highlighted the differential expression of inflammatory proteins (CRP, SAA1, and SAA2) in the volcano plot. Notably, P02741 (CRP) and P0DJI9 (SAA2) showed differential expression across all three groups, while P0DJI98 (SAA1) did not exhibit differential expression between the chronic Brucellosis group and the healthy controls. This suggests that CRP and SAA2 may hold diagnostic value in monitoring the progression of Brucellosis to chronic infection.

To further describe the expression patterns of the 42 common differentially expressed proteins, we generated a heatmap. Clustering analysis was performed on both the samples and proteins within the heatmap. The results revealed that the samples were effectively grouped into three main clusters: acute Brucellosis, chronic Brucellosis, and healthy controls, as shown in **Figure 2B**. Notably, the expression patterns of these 42 proteins aligned with the results of the differential analysis. In addition, we analyzed the expression levels of eight acute-phase proteins across the three groups. The findings demonstrated that while these proteins were elevated during the acute phase of Brucellosis, CRP, FGB, LYZ, and AAT did not return to the baseline levels observed in healthy controls in chronic patients. This suggests the persistence of long-term inflammation in chronic Brucellosis, as illustrated in **Figure 2C**.

To further investigate the biological processes and enriched pathways involved in the chronic progression of Brucellosis, we conducted Gene Ontology (GO) and Kyoto Encyclopedia of Genes and Genomes (KEGG) enrichment analyses on the 120 commonly differentially expressed proteins. The GO analysis (**Figure 2D**) results show that several significantly enriched terms in the biological process (BP) category are primarily related to immune cell migration and proliferation, as well as cell movement and adhesion; In the cellular component (CC) analysis, “extracellular exosome” is a significantly enriched component; In the molecular function (MF) category, “protein binding” is a significantly enriched term. The KEGG pathway analysis (**Figure 2E**) revealed that the differentially expressed proteins were primarily involved in pathways including the VEGF signaling pathway, B cell receptor signaling pathway, Fc gamma R-mediated phagocytosis, MAPK signaling pathway, and natural killer cell-mediated cytotoxicity. Additionally, we present the results of the Brucellosis group analysis with the healthy control group in **Supplementary Figure 1**.

### 3.2 Identifying co-expressed protein modules associated with clinical indicators in brucellosis patients using WGCNA

**Figure 3:**
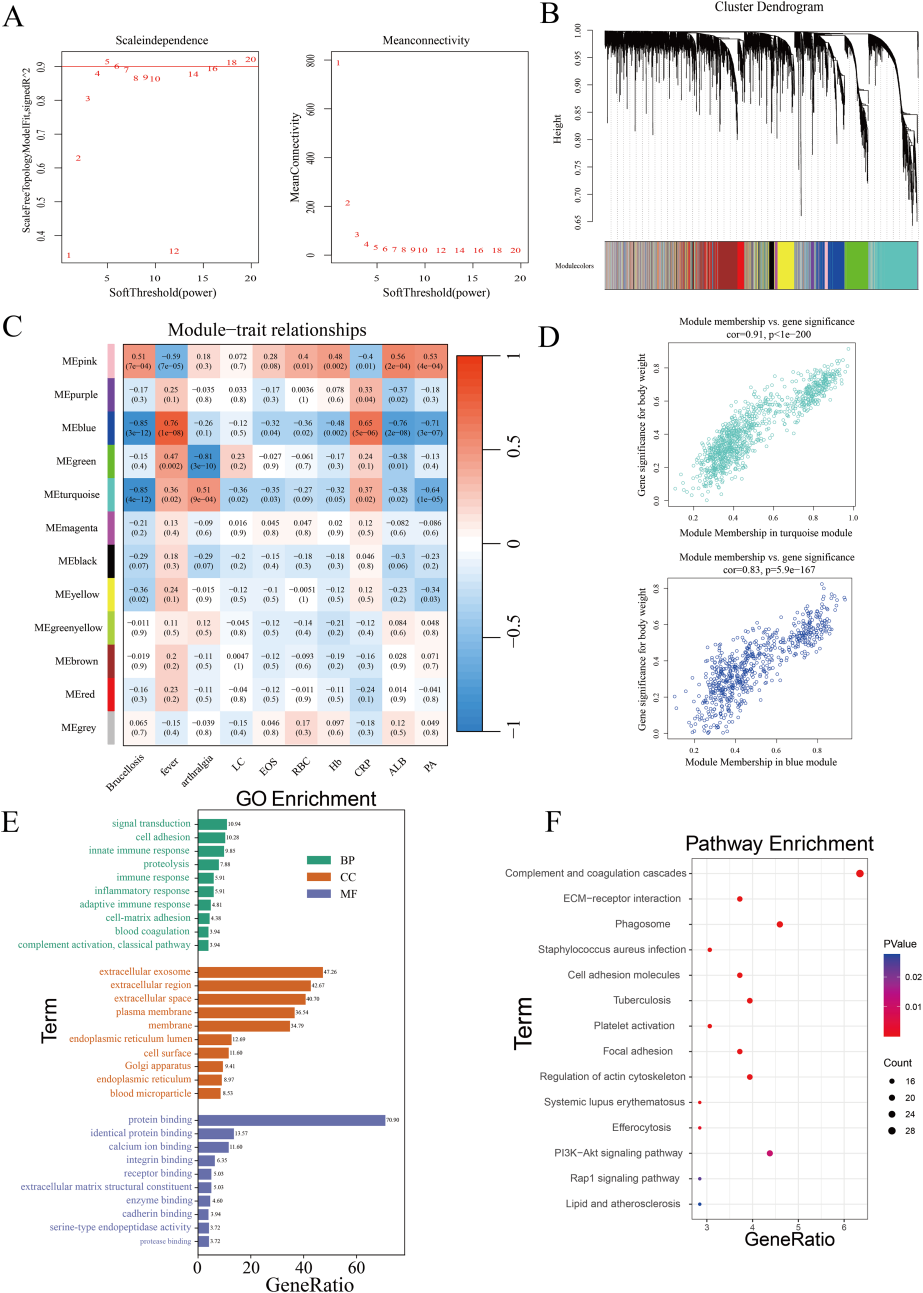
Identification of co-expressed protein modules using WGCNA. **(A)** β=5 was selected to establish a scale-free network. **(B)** Dendrogram of clustering. **(C)** Heatmap showing the correlation between gene modules and clinical features. (LC, Lymphocytes; EOS, Eosinophils; RBC, Red Blood Cells; Hb, Hemoglobin; CRP, C-Reactive Protein; ALB, Albumin; PA, Prealbumin.) **(D)** Correlation of the two most highly correlated modules. **(E)** GO analysis of modules. **(F)** KEGG analysis of modules.

To identify co-expressed protein modules, we applied the Weighted Gene Co-expression Network Analysis (WGCNA) method to construct a weighted co-expression network from the expression data of 5060 proteins. Cluster analysis was performed across three distinct sample groups, revealing clear differentiation among them, with no outliers or abnormal samples identified (**Supplementary Figure 2A**). As shown in **Figure 3A**, a scale-free topology criterion with a fitting index of 0.9 indicated that the minimum soft thresholding power required to achieve a scale-free network was 5. Therefore, a soft thresholding power of 5 was selected as the optimal parameter for subsequent analysis. This process led to the identification of 12 distinct modules (**Figure 3B**), including 11 co-expressed protein modules and one gray module (MEgray), which was excluded from further analysis. The heatmap displaying inter-module correlations is provided in **Supplementary Figure 2B**. To assess the relationship between the identified modules and clinical traits, we utilized the clinical symptoms and laboratory indicators of Brucellosis patients. A heatmap was generated to illustrate the correlations between these traits and the protein modules (**Figure 3C**). Detailed clinical information for the patients is provided in **Supplementary Table 1**, while the full heatmap showing the correlations can be found in **Supplementary Figure 2C**. The analysis revealed that the blue module was positively correlated with the clinical symptom of fever, the blue and turquoise modules were negatively correlated with Brucellosis infection, and the green module was negatively correlated with the clinical symptom of arthralgia.

Subsequently, we analyzed the significance of proteins in the turquoise module related to Brucellosis infection and in the blue module associated with fever, as shown in **Figure 3D**. The top 20 proteins with the highest connectivity within the turquoise and blue modules were selected and imported into Cytoscape software for further analysis. The network relationships of these top 20 hub proteins in both the turquoise and blue modules are visualized in **Supplementary Figure 2D**. These characteristic proteins may play critical biological roles in Brucellosis infection and its associated clinical symptoms.

Finally, we performed Gene Ontology (GO) and Kyoto Encyclopedia of Genes and Genomes (KEGG) pathway analyses on all proteins in the turquoise and blue modules. The GO analysis results (**Figure 3E**) show that in the biological process (BP) category, multiple significantly enriched terms are primarily related to immune response and cell function regulation. In the cellular component (CC) analysis, “extracellular exosome” is a significantly enriched component. In the molecular function (MF) category, “protein binding” is the significantly enriched term. The results of the KEGG enrichment analysis (**Figure 3F**) showed that these proteins participate in pathways such as ECM-receptor interaction, complement and coagulation cascades, PI3K-Akt signaling, phagosome formation, and tuberculosis.

### 3.3 Identification of key proteins for the diagnosis of brucellosis using machine learning

**Figure 4:**
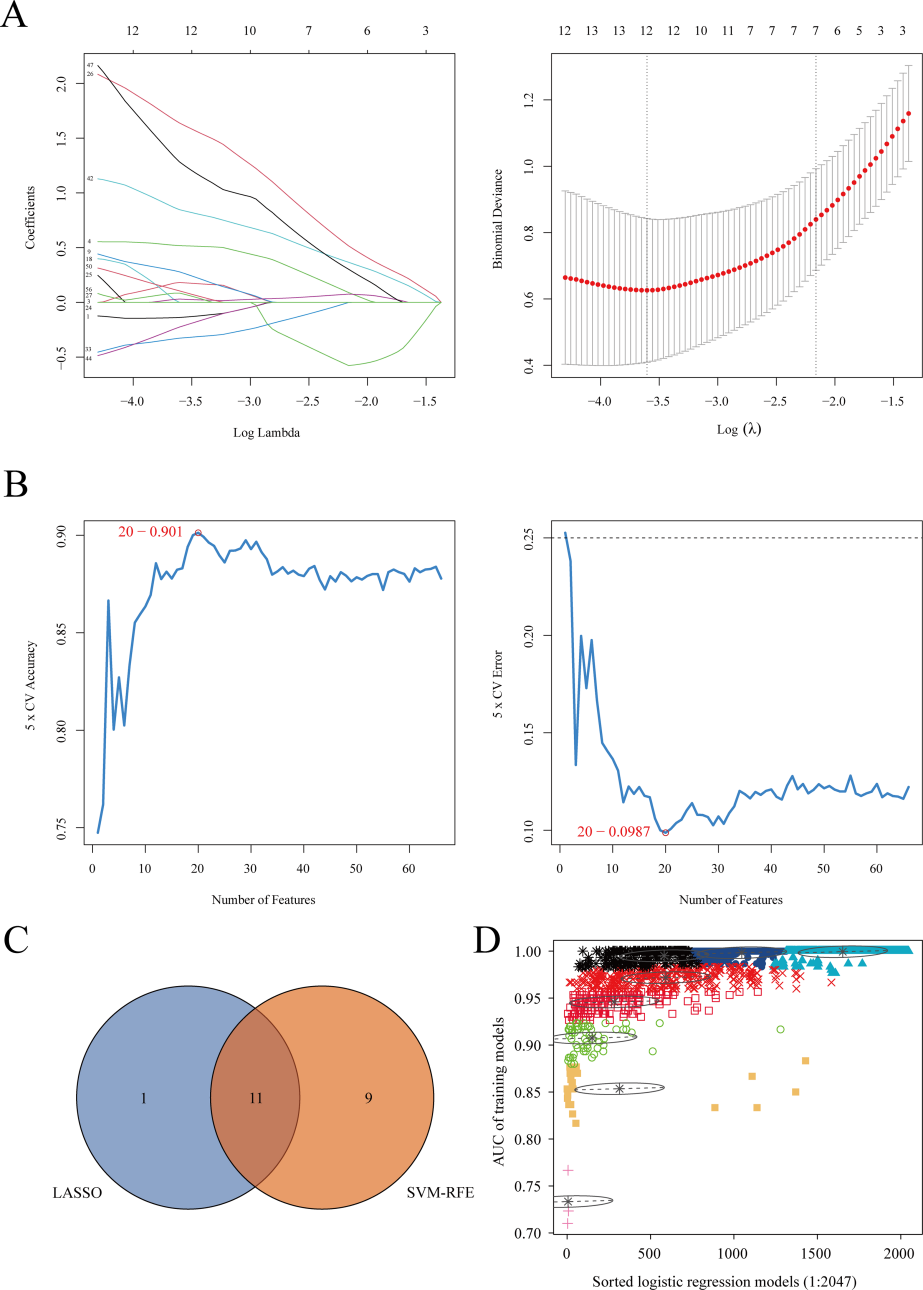
Machine learning algorithm selection of potential protein biomarkers for brucellosis diagnosis. **(A)** Key proteins were selected using the Lasso model, with the number of proteins at the lowest point of the curve (n = 12) considered the most suitable for the Lasso model. **(B)** Key proteins were selected using the SVM-RFE model, with 20 proteins included. **(C)** Venn diagram showing proteins common to both algorithms. **(D)** The GMM algorithm was used for the 11 common proteins, and the combination with the optimal AUC was selected as the potential biomarker.

We cross-referenced 42 commonly differentially expressed proteins, 120 proteins potentially related to chronicity, and 227 proteins associated with the acute phase from differential analysis with co-expressed proteins in the blue and turquoise modules of WGCNA to identify key proteins. This process yielded a total of 229 cross-identified proteins. After retrieving data from the UniProt database (https://www.uniprot.org), we excluded unreviewed immunoglobulin complexes and proteins with missing expression data. Ultimately, 69 proteins were retained for subsequent machine learning analyses to identify the optimal combination of diagnostic markers (**Supplementary Table 2**). Two machine learning algorithms were applied: LASSO regression analysis and the SVM-RFE algorithm, to select potential diagnostic proteins. LASSO regression identified 12 proteins (**Figure 4A**), while the SVM-RFE method identified 20 proteins (**Figure 4B**). A Venn diagram was used to illustrate the 11 common key proteins selected by both methods (**Figure 4C**). Finally, the 11 key proteins were input into the GMM algorithm, and the top 6 protein combinations with the highest diagnostic values were obtained from the purple module (**Figure 4D**). The identified proteins and their respective UniProt IDs are as follows: A0A1S5UZ24 (Macrophage receptor), A0A6Q8PFK8 (Heat shock protein family B (small) member 1), B9EJA8 (Mannose receptor, C-type 1-like 1), P00966 (Argininosuccinate synthase), P05362 (Intercellular adhesion molecule 1), and P0DJI9 (Serum amyloid A-2 protein).

### 3.4 Enzyme-linked immunosorbent assay validation

We validated the selected proteins using ELISA kits and found that ASS1 did not decrease in the acute phase, as indicated by the proteomics analysis. No statistically significant differences were observed in ASS1 levels across the three groups. In contrast, the results for CD206, MARCO, SAA2 and VCAM-1 were consistent with the proteomics findings. HSPB1 was elevated in both the acute and chronic phases, which did not align with the proteomics data but still exhibited a notable difference (**Supplementary Figure 3A**). Based on these discrepancies, we excluded ASS1 from further analysis and combined the remaining five proteins, which demonstrated strong diagnostic efficiency for brucellosis (**Supplementary Figure 3B**).

## 4 Discussion

In this study, we utilized Orbitrap-DIA mass spectrometry technology combined with bioinformatics methods to perform a proteomic analysis of serum samples from patients with acute and chronic brucellosis, as well as healthy controls. The primary goal was to identify potential serum biomarkers associated with *Brucella* infection. Our analysis revealed a set of differentially expressed proteins that show promise for aiding in the diagnosis of brucellosis. Furthermore, these proteins may offer valuable insights into the pathogenesis of the disease, providing a foundation for future research efforts.

Through differential protein analysis, we identified 42 proteins that were differentially expressed across all three groups, 120 proteins associated with chronic brucellosis, and 227 proteins linked to the acute phase. Using weighted gene co-expression network analysis (WGCNA), we identified two co-expression protein modules that were associated with clinical features of brucellosis. Following machine learning algorithm selection, we identified 6 proteins with superior diagnostic efficacy. After final validation using ELISA, five proteins were confirmed as potential diagnostic biomarkers, demonstrating strong combined diagnostic capability. These five proteins show significant diagnostic value, especially in cases where acute-phase patients have not yet developed antibodies, leading to false-negative results, or when delays in blood culture processing complicate diagnosis. In addition, for clinical patients with low antibody titers but presenting symptoms, these protein biomarkers may provide valuable diagnostic guidance. In the next section, we will discuss the potential roles of these five proteins in *Brucella* infection.

The Macrophage Receptor with Collagenous Structure (MARCO), represented by the protein ID A0A1S5UZ24, is a member of the class A scavenger receptor family and plays a key role in the innate antimicrobial immune response. Under normal conditions, it is primarily expressed on tissue macrophages, particularly in the spleen and lymph nodes (Kraal et al., 2000). *Brucella*, a facultative intracellular pathogen, primarily resides within the host’s macrophages. The pathogen-associated molecular patterns (PAMPs) of *Brucella*, such as lipopolysaccharides (LPS), can be recognized and bound by pattern recognition receptors (PRRs) on macrophages, initiating innate immune responses (Yu et al., 2024). MARCO has several functions, including adhesion, migration, phagocytosis, and cytokine secretion, all of which involve complex interactions among various immune cells. As such, MARCO plays a crucial role in triggering immune responses, bridging innate and adaptive immunity, and helping to eliminate pathogens (Zhou et al., 2024). MARCO can also interact with other PRRs, such as Toll-like receptors (TLRs). The binding of LPS-LBP-CD14 complexes to TLR4 induces the upregulation of MARCO on macrophages, enhancing their phagocytic function (Chen et al., 2010). This may explain the upregulation of MARCO expression observed in acute-phase patients in this study (**Supplementary Figure 3C**). Additionally, research shows that *Brucella* ‘s LPS binds to class A scavenger receptors, promoting bacterial internalization into macrophages (Kim et al., 2004). This interaction may contribute to the establishment of chronic infection and immune evasion by *Brucella*.

Mannose Receptor, C-Type 1-Like 1 (MRC1L1), also known as CD206 (protein ID: B9EJA8), is primarily expressed on the surface of activated macrophages. It recognizes various polysaccharide components of the cell wall, including mannose, lipopolysaccharides (LPS), and capsular polysaccharides (Cummings, 2022). As a pattern recognition receptor (PRR), CD206 plays a crucial role in both innate and adaptive immunity by enhancing the phagocytic and antigen-presentation capabilities of macrophages (Kerrigan and Brown, 2009). In addition to its membrane-bound form, CD206 can be cleaved by matrix metalloproteinases (MMPs) (Martínez-Pomares et al., 1998) and released into the extracellular space as a soluble form known as soluble mannose receptor (sMR). Research indicates that pathogen-specific stimulation can trigger shedding of sMR, which can then be detected in serum (van der Zande et al., 2021). Elevated levels of sMR have been observed in patients with various inflammatory conditions, such as pneumonia (Kazuo et al., 2019) and sepsis (Kjærgaard et al., 2014), suggesting that sMR could serve as a novel biomarker for inflammation. However, its exact role in these diseases remains unclear. One study has proposed that sMR can activate pro-inflammatory macrophages (M1 phenotype) by binding to CD45 on macrophages (Embgenbroich et al., 2021). In our study, we observed elevated sMR levels in patients during the acute phase (**Supplementary Figure 3D**), indicating that *Brucella* infection may be associated with sMR-induced innate immune responses.

Intercellular Adhesion Molecule-1 (ICAM-1), also known as CD54, with the protein ID P05362, plays a crucial role in the inflammatory response. During inflammation, ICAM-1 is upregulated on a variety of cell types, particularly leukocytes and endothelial cells. This upregulation facilitates leukocyte adhesion to the endothelial cell surface, their transmigration across the vascular wall, and the repair of endothelial cell damage (Haydinger et al., 2023). ICAM-1 expression is induced by a range of inflammatory stimuli, including tumor necrosis factor-alpha (TNF-α), lipopolysaccharide (LPS), interleukin-6 (IL-6) (Caldenhoven et al., 1994), and reactive oxygen species(ROS) (Roebuck et al., 1995). Under normal conditions, ICAM-1 expression on macrophages is relatively low. However, in response to inflammatory stimuli, this expression is markedly upregulated, enhancing macrophage function. Specifically, studies have shown that ICAM-1 upregulation in macrophages enhances their phagocytic activity in response to LPS stimulation (Zhong et al., 2021). In the context of inflammation, ICAM-1, together with vascular cell adhesion molecule-1 (VCAM-1), mediates leukocyte-endothelial interactions, such as adhesion and luminal crawling. This process facilitates the migration of immune cells, including neutrophils and macrophages, across the endothelium (Ren et al., 2023). Upon infection with *Brucella* bacteria, the bacteria adhere to endothelial cells, triggering pro-inflammatory responses and endothelial damage (Togan et al., 2015), This inflammatory response leads to leukocyte infiltration around blood vessels and an increase in the expression of adhesion molecules like ICAM-1 and VCAM-1. In our study, we observed elevated levels of endothelial injury markers, including von Willebrand factor (VWF), VCAM-1, and ICAM-1, in patients with acute and chronic Brucellosis (**Supplementary Figure 3E**). Moreover, indicators of platelet adhesion, which are involved in endothelial repair, were reduced due to platelet attrition (**Supplementary Figure 3F**). We speculate that *Brucella* may damage vascular endothelial cells, destabilizing endothelial function. Additionally, this observation may be related to the direct binding of platelets to *Brucella*, forming complexes that influence *Brucella* infection of macrophages. Thrombocytopenia could thus contribute to the progression of Brucellosis and the persistence of the infection (Trotta et al., 2018).

Heat Shock Protein Beta-1 (HSPB1), also known as HSP27, with the protein ID A0A6Q8PFK8, plays a pivotal role in antioxidation, anti-apoptosis, and the innate immune response. In this study, we observed that HSPB1 was significantly upregulated in patients with chronic Brucellosis compared to those with acute Brucellosis and healthy controls. KEGG enrichment analysis further revealed that both P15153 and A0A6Q8PFK8 were significantly enriched in the VEGF signaling pathway. Previous research has shown that HSPB1 can be released from endothelial cells and inhibit angiogenesis by interacting with vascular endothelial growth factor (VEGF) (Lee et al., 2012). Additionally, extracellular HSPB1 can exert pro-inflammatory effects by upregulating the expression of monocyte chemoattractant protein (MCP-1) and intercellular adhesion molecule (ICAM-1) on human coronary artery endothelial cells. This occurs through activation of the NF-κB pathway following interaction with Toll-like receptors TLR-2 and TLR-4 (Jin et al., 2014). Moreover, overexpression of HSPB1 enhances its antioxidative properties, which can help prevent cardiovascular ischemic injury (Vidyasagar et al., 2012). The Rac family small GTPase (P15153) has been shown to increase reactive oxygen species (ROS) production (Mittal et al., 2014); however, its expression is decreased in chronic phase patients (**Supplementary Figure 3G**). Additionally, the upregulation of superoxide dismutase 1 (SOD1) in chronic phase patients suggests that *Brucella* may facilitate chronic infection by reducing intracellular ROS levels, thus inhibiting apoptosis (Li et al., 2016). Oxidative stress, which results from increased ROS levels, significantly induces HSPB1 expression (Singer et al., 2022). HSPB1 can act as an antioxidant by reducing intracellular iron levels, thereby mitigating ROS accumulation and exerting anti-apoptotic effects (Arrigo et al., 2005).

Serum amyloid A-2 (SAA2), identified by the protein ID P0DJI9, is a member of the apolipoprotein serum amyloid A family, primarily produced by the liver as an acute-phase reactant. During inflammation and tissue damage, both SAA1 and SAA2 are upregulated, with varying degrees of elevation observed during the acute and chronic phases of diseases. Currently, research on SAA2 is limited, with most studies focusing primarily on SAA1. SAA1 is known to upregulate the expression of various inflammatory mediators in multiple cell types, including leukocytes, fibroblasts, and endothelial cells. These mediators include cell adhesion molecules, cytokines, chemokines, matrix-degrading proteinases, reactive oxygen species (ROS), and pro-angiogenic molecules (Abouelasrar Salama et al., 2020). In this study, we found that in patients with chronic Brucellosis, the levels of SAA2 were elevated compared to SAA1, while the expression levels of SAA1 during the chronic phase were comparable to those of healthy individuals. Finally, we created a diagram illustrating the potential mechanisms of these biomarkers as diagnostic tools for Brucellosis infection and its chronic progression (**Supplementary Figure 4**).

Our study has several limitations. First, the sample size is relatively small, which may have caused low-abundance proteins to be overlooked. Second, the sample types are homogeneous, consisting solely of serum from patients. However, our study population includes individuals in the acute phase with NeuroBrucellosis and chronic phase patients with joint pain symptoms. Future studies incorporating other bodily fluids, such as cerebrospinal or synovial fluid, could help to further elucidate the pathogenic mechanisms and biomarkers of Brucellosis in these specific conditions. Additionally, immunoglobulin fragments in the serum proteome were excluded from our analysis, which may have led to the omission of potential biomarkers. Most importantly, we are unable to pinpoint which organs or cell types are responsible for these changes. Despite these limitations, the identified proteins demonstrate promising diagnostic potential for differentiating acute, chronic, and healthy groups, providing a basis for future studies into the detailed pathogenic mechanisms of Brucellosis.

## Data Availability

The datasets presented in this study can be found in online repositories. The names of the repository/repositories and accession number(s) can be found below: http://www.proteomexchange.org/,PXD051999.
