# Supplementary material for: Analysis and validation of serum biomarkers in brucellosis patients through proteomics and bioinformatics
Source: Front Cell Infect Microbiol. 2025 Jan 13;14:1446339. doi: 10.3389/fcimb.2024.1446339 (PMC11769985; doi:10.3389/fcimb.2024.1446339)
Supplement: Supplementary file 3 [file DataSheet3.docx]

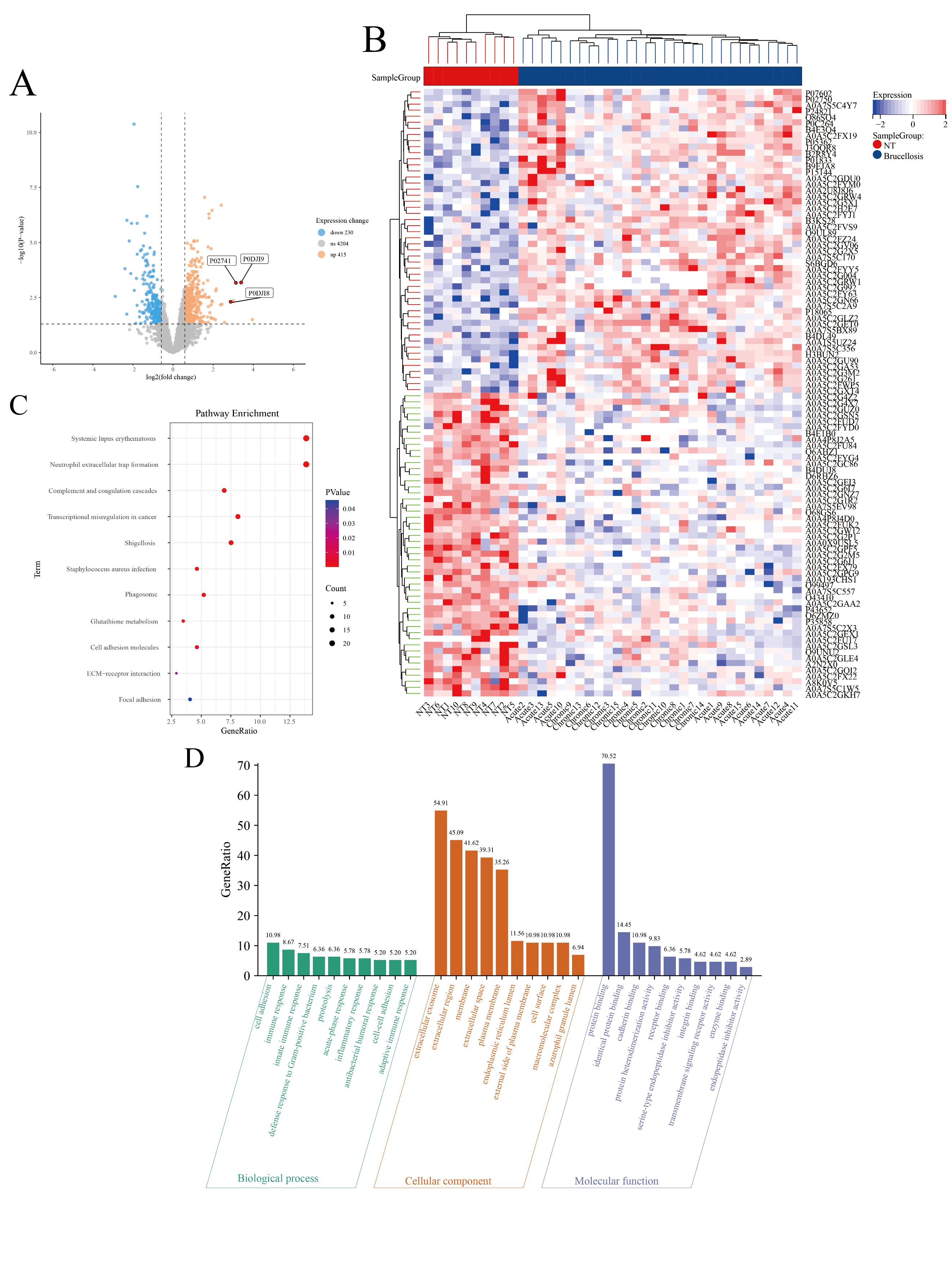


Supplementary Figure 1: **(A)** Volcano plot showing differential protein expression between the Brucellosis group and healthy controls. **(B)** Heatmap of the top 50 differentially expressed proteins between the Brucellosis group and healthy controls. **(C)** Kyoto Encyclopedia of Genes and Genomes (KEGG) analysis of differentially expressed proteins. **(D)** Gene Ontology (GO) analysis of differentially expressed proteins.


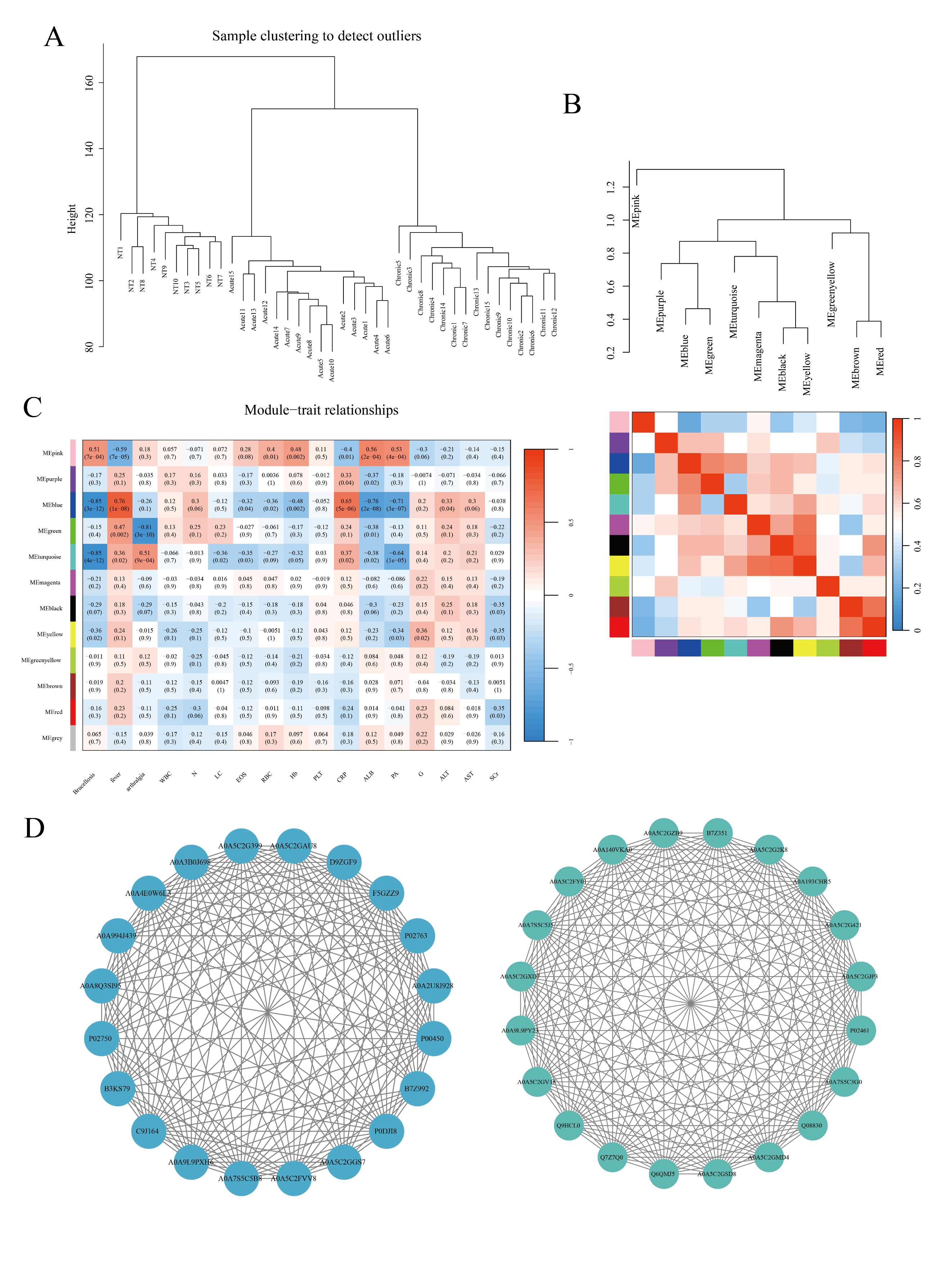


Supplementary Figure 2: **(A)** Cluster analysis of samples from three groups. **(B)** Heatmap showing the correlation between modules and module clustering. **(C)** Heatmap displaying the correlation between comprehensive clinical information and modules. (**LC**: Lymphocytes, **EOS**: Eosinophils, **RBC**: Red Blood Cells, Hb: Hemoglobin, **CRP**: C-Reactive Protein, **ALB**: Albumin, **PA**: Prealbumin, **WBC**: White Blood Cell, **N**: Neutrophils, **ALT**: Alanine Aminotransferase, **AST**: Aspartate Aminotransferase, **Scr**: Serum Creatinine, **G**: Globulin) **(D)** Interaction network diagram of the top 20 proteins in the blue and turquoise modules.


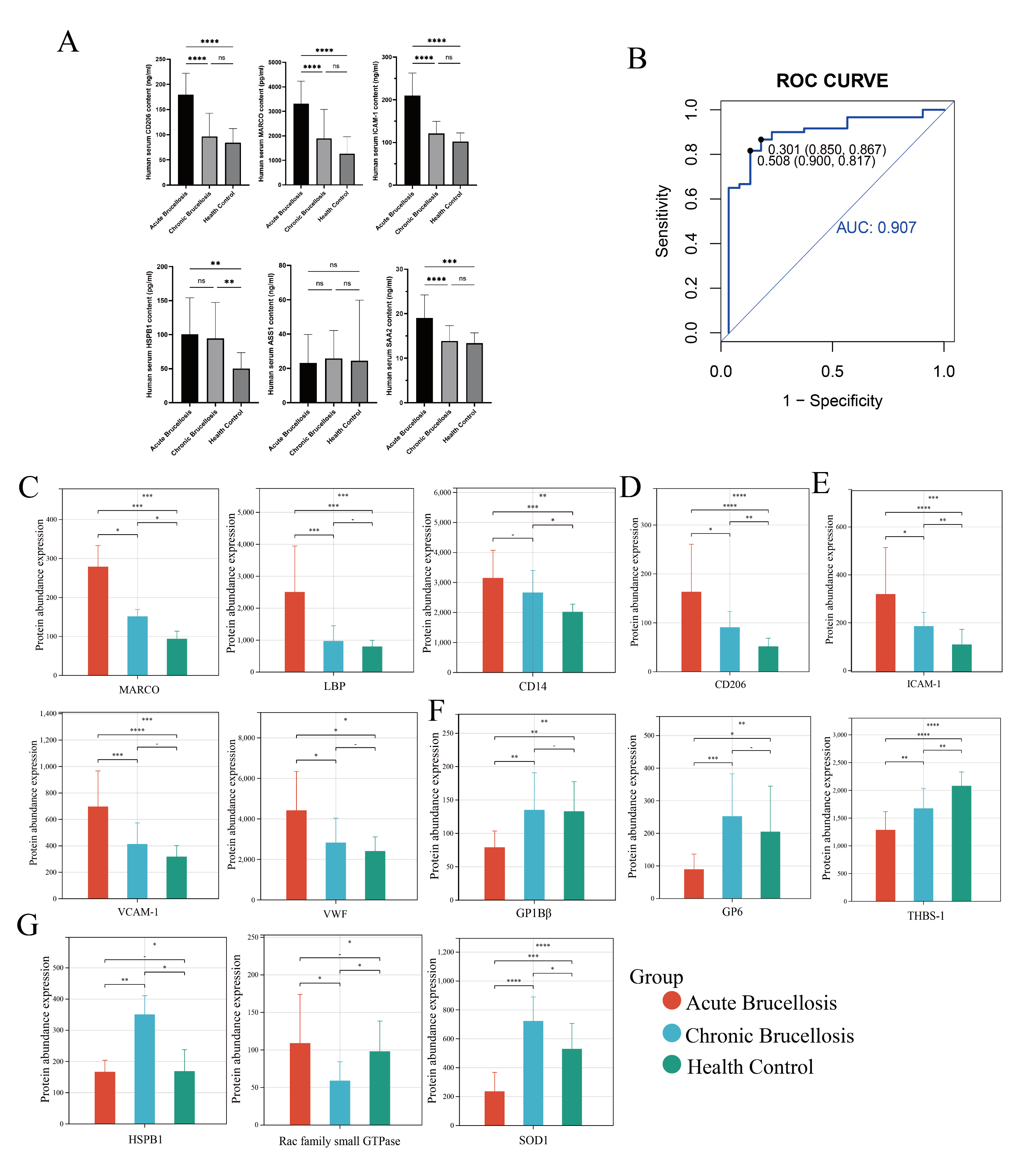


Supplementary Figure 3: **(A)** ELISA verified the actual expression of the six proteins. (Test method: **one-way ANOVA**) **(B)** ROC curve of the combination of five proteins in diagnosis. **(C)** Relative protein abundance of MARCO, LBP, and CD14. (**MARCO**: Macrophage Receptor with Collagenous Structure, **LBP**: Lipopolysaccharide Binding Protein, **CD14**: Monocyte Differentiation Antigen CD14.) **(D)** Relative protein abundance of CD206.(**CD206**: Mannose receptor, C-type 1-like 1.) **(E)** Relative protein abundance of markers associated with vascular endothelial activation and injury.(**ICAM-1**: Intercellular Adhesion Molecule-1, **VCAM-1**:Vascular Cell Adhesion Molecule-1, **VWF**: Von Willebrand Factor.) **(F)** Relative protein abundance of markers associated with platelet adhesion and activation.(**GP1Bβ**: Glycoprotein Ib Beta Chain, **THBS-1**: Thrombospondin-1, **GP6**: Glycoprotein VI.) **(G)** Relative protein abundance of three markers associated with reactive oxygen species (ROS) changes.( **HSPB1**:Heat Shock Protein Beta-1, **SOD1**:Superoxide Dismutase 1.)


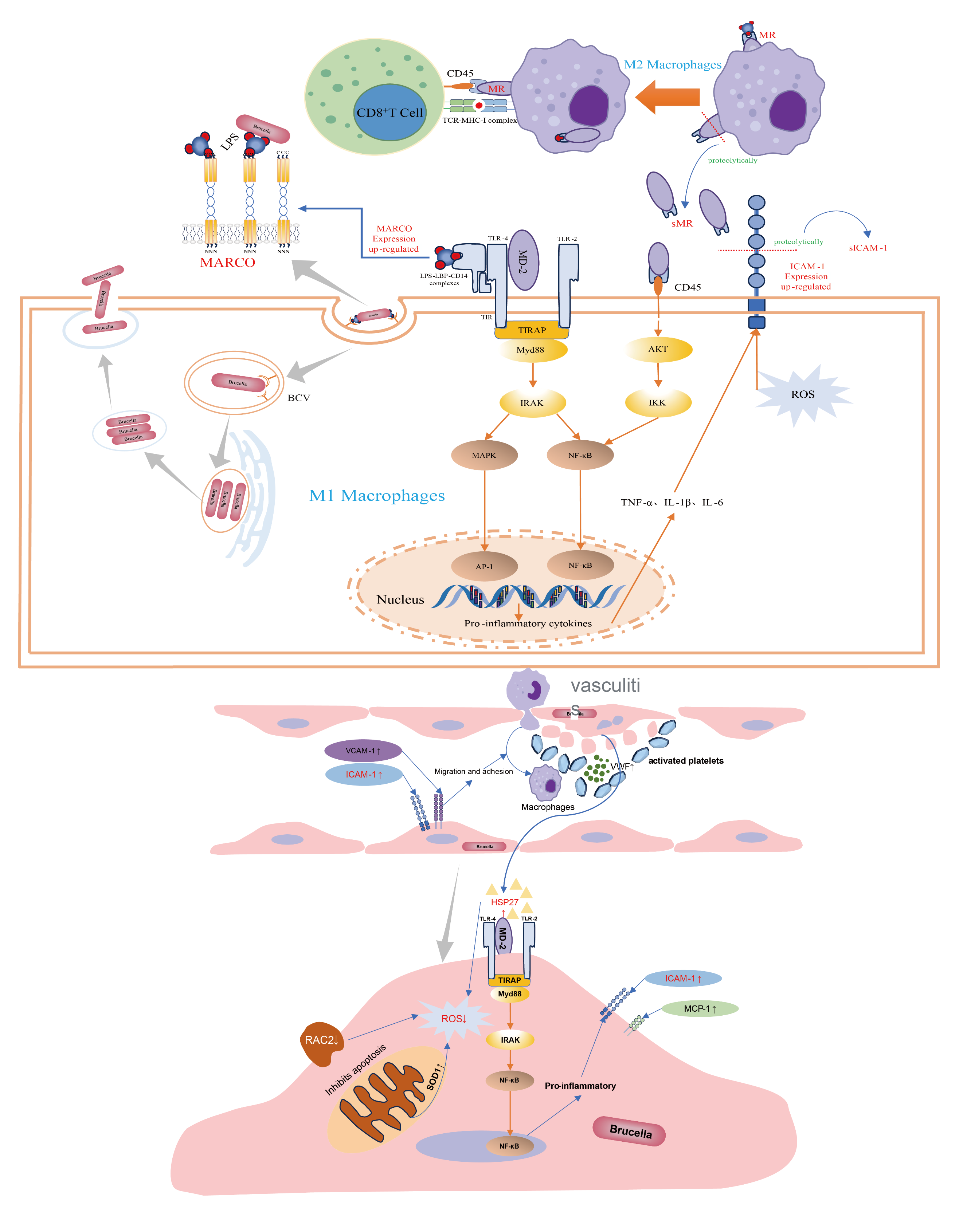


Supplementary Figure 4: Possible mechanisms of action of diagnostic markers of brucellosis in macrophages and endothelial cells.
